# Effects of participatory ‘A’rt-Based Activity On ‘Health’ of Older Community-Dwellers: results from a randomized control trial of the Singapore A-Health Intervention

**DOI:** 10.3389/fmed.2023.1238562

**Published:** 2023-12-21

**Authors:** Andy Hau Yan Ho, Stephanie Hilary Xinyi Ma, Michael Koon Boon Tan, Ram Bajpai, Shannon Shuet Ning Goh, Gabriellia Yeo, Alicia Teng, Yilin Yang, Kévin Galéry, Olivier Beauchet

**Affiliations:** ^1^Action Research for Community Health Laboratory, Psychology Program, School of Social Sciences, Nanyang Technological University, Singapore, Singapore; ^2^Lee Kong Chian School of Medicine, Nanyang Technological University, Singapore, Singapore; ^3^Palliative Care Centre for Excellence in Research and Education, Singapore, Singapore; ^4^Lab4Living, Culture and Creativity Research Institute, Sheffield Hallam University, Sheffield, United Kingdom; ^5^School of Medicine, Keele University, Newcastle-under-Lyme, United Kingdom; ^6^National Gallery Singapore, Community and Access, Singapore, Singapore; ^7^Research Centre of the Geriatric University Institute of Montreal, Montreal, QC, Canada; ^8^Departments of Medicine and Geriatrics, Faculty of Medicine, University of Montreal, Montreal, QC, Canada

**Keywords:** participatory arts, museum, social prescribing, frailty, wellbeing, older adults, randomized control trial, Singapore

## Abstract

**Introduction:**

The practice of participatory art has been found to support the promotion, prevention, and management of health across the lifespan. However, clinical trials investigating the benefits of creative activities curated with and conducted in museums among older adults in East Asia remains limited.

**Methods:**

The current research utilized a single-site, open-label randomized control trial (RCT) to evaluate a standardized Participatory ‘A’rt-Based Activity On ‘Health’ of Older Community-Dwellers – the Singapore A-Health Intervention. Outcome measures include frailty as assessed by the Centre of Excellence on Longevity Self-administered Questionnaire, wellbeing as assessed by the Warwick-Edinburgh Mental Wellbeing Scales, and quality of life as assessed by the EuroQol-5D. 112 participants aged 60 and above were randomized into the intervention group (*n* = 56) or an inactive control group (*n* = 56). Participants completed four standardized online self-administered assessments at baseline, 5-week, 9-week and 12-week follow-up during the intervention period.

**Results:**

Linear mixed model analyses revealed no statistically significant differences between the intervention group and control group for all outcome measures. However, within the intervention group, a consistent significant reduction in frailty was observed across time from baseline to 9 weeks (MD −0.44, 95% CI −0.85 to −0.039, *p* = 0.032), 5-weeks to 9-weeks (MD −0.64, 95% CI −1.03 to −0.24, *p* = 0.002), and 5-weeks to 12-weeks (MD −0.51, 95% CI −0.91 to −0.10, *p* = 0.014). Moreover, the post-test mean wellbeing score in the intervention group significantly improved over time at 9-weeks (MD 1.65, 95% CI 0.09 to 3.22, *p* = 0.039) and 12-week (MD 2.42, 95% CI 0.67 to 4.16, *p* = 0.006) as compared to baseline scores.

**Discussion:**

The findings demonstrate the potential of a structured art and museum-based intervention as a resource for promoting health among aging populations. Such benefits transcend social, cultural, and societal contexts.

**Clinical trial registration:**

ClinicalTrial.gov, NCT05945589.

## 1 Introduction

### 1.1 Background

Population aging continues to be global health challenge. The World Health Organization anticipates that by 2050, the world’s population of people aged above 60 years old will double, while those aged 80 years and above will triple (1). The prevalence of longstanding age-related conditions such as worsening physical, social, and mental health is expected to increase exponentially with a rapidly aging population (2–4). This will inevitably cause a surge in the demand for health services, and therefore a pressing need to innovate and develop creative solutions to assuage the strain on the health care system. There are many forms of non-pharmacological interventions designed to complement current medical interventions with physical activity associated with reductions in frailty (5, 6), however, the practice of participatory art across diverse settings has demonstrated its effectiveness in promoting, preventing, and managing health conditions across the life span (7). Participatory arts engagement can be categorized into two primary areas: active involvement which involves the act of creating or performing art, and passive consumption, which involves attending cultural activities, fostering esthetic appreciation, emotional, and sensory stimulation (8). While there are many forms of art, including visual arts, performing arts, literary arts, cultural heritage, and film as defined by scholars and art councils (8–10), participatory arts activities often encompass a combination of art forms, blurring the boundaries between active participation and passive consumption. Examples of active engagement in visual arts activities could include painting, sculpting, and craftwork while passive engagement involves visiting art fairs or guided museum tours. These activities could be implemented by a wide variety of facilitators such as artists, educators, museum docents, and trained researchers (11–13). Specifically for the older population, studies on visual arts and museum-based programs signaled its effectiveness as a low-risk intervention for the management of psychological symptoms and cognitive functioning (13, 14). However, systematic reviews conducted on these interventions revealed that most studies assessed its effects on mental and emotional wellbeing (12, 15–17), and its effects on health conditions among older adults, particularly in the East Asian context, remains to be investigated. In addition to visual art interventions, there is a growing interest and utilization of the museum artifacts for health promotion among the older population. Common types of programs included reminiscence, object-oriented, art-based, storytelling, and lectures curated for the older audiences (18). Similarly, these programs assessed emotional and social outcomes among their participants, but rarely on physical health outcomes (19, 20).

In 2015, the Centre of Excellence on Longevity of McGill University Canada, together with the Montreal Museum of Fine Arts, developed the ‘Participatory Art-Based Activity on Health of Older Community-Dwellers’ (i.e., A-Health), with the goal to standardize a robust 12-week framework of art intervention for health enhancement. Findings from a pre-post single arm pilot study of A-Health indicated that curated, sustained and professionally led museum-based art activity can improve quality of life, psychological wellbeing, and health conditions of older adults in Montreal (21, 22). The success of this pilot study has led to the empirical expansion of A-Health via an international randomized control trial (RCT) for testing its effectiveness in health enhancement among older adults of different society and cultural groups. The international RCT involved multiple centers (i.e., museums/galleries) in various countries and had each developed a culturally unique participatory art-based activity program that adhere to the A-Health framework to facilitate parallel data collection and international data comparison.

The current research adopted the standardized 12-week Montreal A-Health participatory art framework with culturally specific modifications that is suitable for the Singaporean context (i.e., Singapore A-Health Intervention). The modifications include adjustments to the visual art activities offered, as well as the selection of the artefacts for the museum tours to align the intervention with the cultural background and historical context of Singapore. The objective of the study was to evaluate the Singapore A-Health Intervention’s effectiveness in health and wellness promotion among a sample of Singaporean older adults via a randomized control trial. It was hypothesized that participants in the intervention group will experience greater wellbeing, quality of life and reduced frailty as compared to those assigned to the control group. In addition, the insights gained could foster practice and knowledge transfer to accelerate creative and healthy aging in the local community. The Singapore A-Health Intervention was jointly designed by the Action Research for Community Health (ARCH) Laboratory at Nanyang Technological University (NTU), Lab4Living at Sheffield Hallam University, and National Gallery Singapore (the Gallery).

## 2 Methodology

### 2.1 Study design and participants

**Figure 1:**
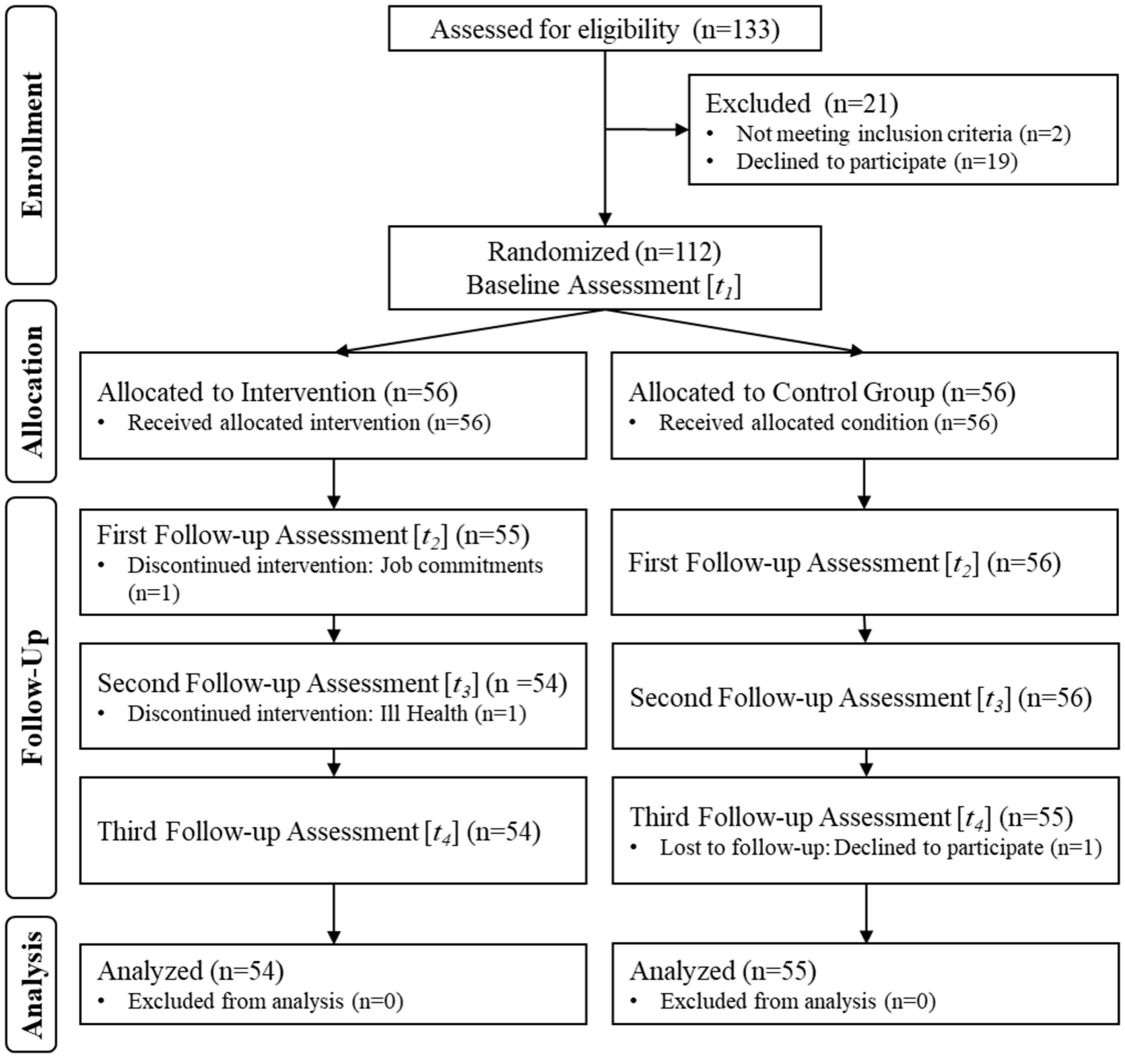
CONSORT flow diagram and study procedures.

The study adopts a participatory action research paradigm and a single-site, open-label randomized control trial (RCT) design to develop and examine the effect of a standardized 12-week museum-based participatory art activity on health condition, wellbeing, and quality of life in older community dwellers (ClinicalTrial.gov, ID: NCT05945589, Institutional Review Board (IRB) Approval: IRB-2020-02-005). Inclusion criteria include community dwelling older adults aged 60 and above, who are fluent in English (the most spoken language among the resident population) and had internet access to complete the online psychometric assessments. Participants who were not able to provide informed consent or were diagnosed with mental health conditions were excluded from the study. Interested participants were required to declare if they had a formal diagnosis of mental health conditions before proceeding with the registration and were asked about their mental health status during the audio or video call with the research team. Participants may also be screened for cognitive acuity using the Mini-Mental State Examination (MMSE) if necessary (23). Eligible participants were recruited through open and rolling recruitment at the museum, partnering Senior Activities Centers (SAC) in Singapore, as well as social media platforms. The choice of social media platforms was based on the existing following of older adults on these accounts. The CONSORT flow diagram is illustrated in Figure 1.

### 2.2 Randomization

Simple randomization was administered by one research team member using an automated randomizer. Consenting participants were randomly assigned a number, where 1 = Intervention group, and 2 = Control group. The randomization outcomes were only revealed to participants and other members of the research team after completion of the baseline assessment to minimise bias.

### 2.3 Procedures

**Figure 2:**
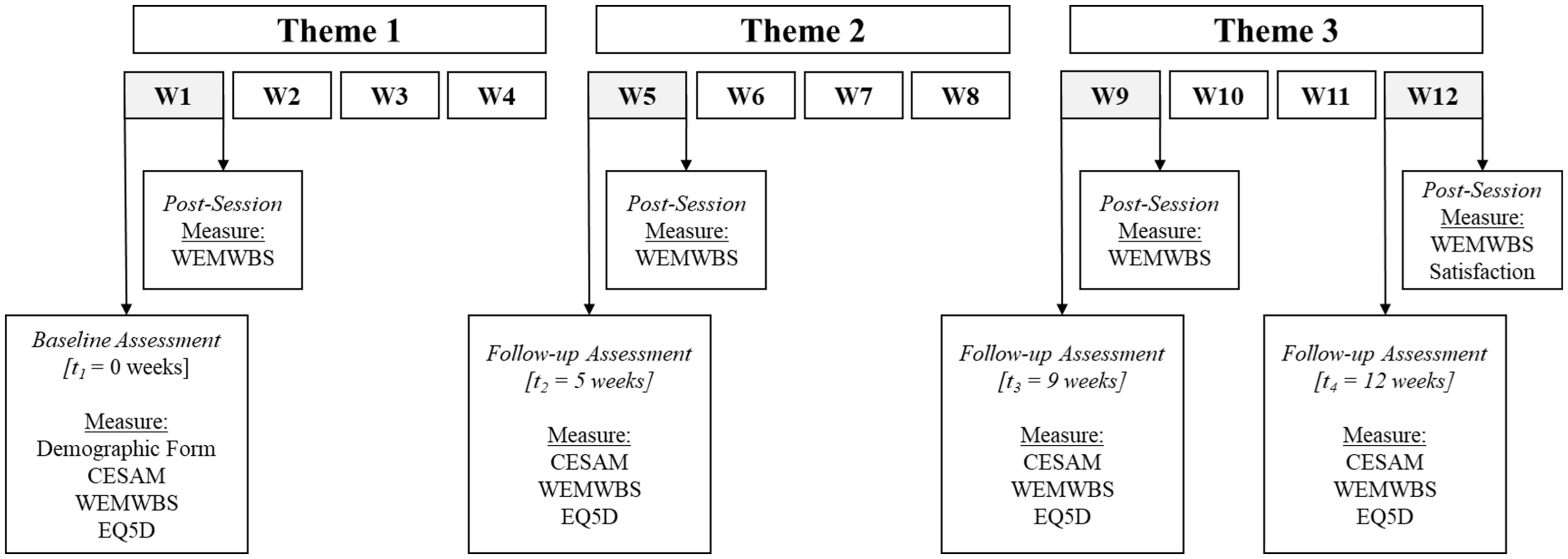
Outcome measures administered to intervention group participants. CESAM, The Centre of Excellence Self-AdMinistered Questionnaire; WEMWBS, Warwick-Edinburgh Mental Wellbeing scale; EQ-5D, EuroQol-5D; Satisfaction, Satisfaction Survey.

Physical and e-copies of the recruitment brochures were distributed through the SACs and on the research and museum team’s Facebook and Instagram pages. Interested individuals could register for the study through the SACs, where they were subsequently referred to the research team. They could also indicate their interest on a Qualtrics sign-up page which included key information about the study as well as the inclusion and exclusion criteria. A member of the research team communicated with interested individuals via phone call or video calls on secured video conferencing channels for screening, informed consent, and baseline assessment. Group placement was revealed only after participants completed the baseline assessment. The study was implemented in two phases. The first phase was a feasibility study where 48 participants were randomized into an intervention group (*n* = 24) or control group (*n* = 24) in March 2021. After refinements in the intervention protocol, the second phase included 62 participants who were randomized into an intervention (*n* = 31) or control group (*n* = 31) in September 2021. Participants in the intervention and control group were asked to complete four standardized online self-administered physical and psychological health assessment at baseline [T1], 5-week [T2], 9-week [T3] and 12-week [T4] follow-up during the intervention period. Intervention group participants were also invited to complete an additional questionnaire on wellbeing immediately after the program at the first week, fifth week, ninth week, and twelfth week of the program (Figure 2). Participants received a SGD$20 (approximately USD$15) incentive for completing each of the four standardized online self-administered questionnaires, totaling SGD$80 (approximately USD$58) per participant. Intervention group participants were invited to participate in a 12-week professionally led participatory art program at the museum. Each week consisted of one 2-h art session, for a total of 24 h of museum tours and participatory art activities. Participants in the control condition were not offered any art-based activities and advised not to participate in concurrent health and art-based interventions during the research period. They were provided with an optional guided museum tour after completing their participation in the 12-week research period.

### 2.4 Intervention design

The 12-week Singapore A-Health Intervention, totaling to 24 h of engagement, adhered to the original intervention’s frequency and duration. The intervention targeted the development and sustained practice of three sequential art domains that aims to impart basic art appreciation skill (formal analysis in art) and art making techniques to participants through engagement with the collection at the museum. Based on an overarching theme of Belonging/Perspectives, the sessions were organized according to thematic domains of Past, Present and Future. The themes are unique to the Singapore program and accentuates the original intervention protocol by drawing inspiration from existing work on life review (24) and life reflection (25). Both of which are narrative techniques structured around life events and themes over life course, where conversations would explore major turning points, such as the impact of major historical events, experiences over life course, meaning, values, and purpose. Life review fosters connection between memories and the meaning of life and appraises an individuals’ capacity to overcome difficult experiences. It contrasts reminiscence or life history, which often provide a more detailed and descriptive account of life events (26). In appreciating these qualities, Past, Present and Future was conceptualized as a conversation eliciting framework. The themes also serve as a unifying and guiding principle for content development.

**Figure 3:**
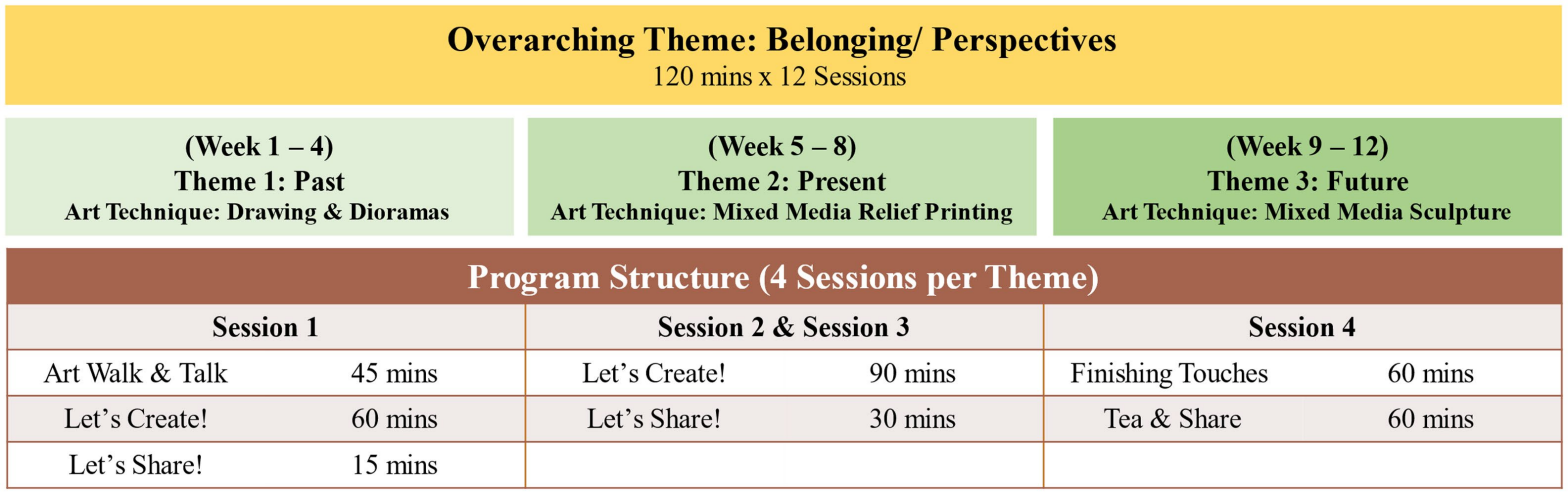
A-Health Singapore intervention structure. Notes on intervention design: (1) The intervention structure was co-developed with the gallery team to curate artworks with the activity structure. (2) Artist and docent training sessions were conducted before the commencement of the program.

**Figure 4:**
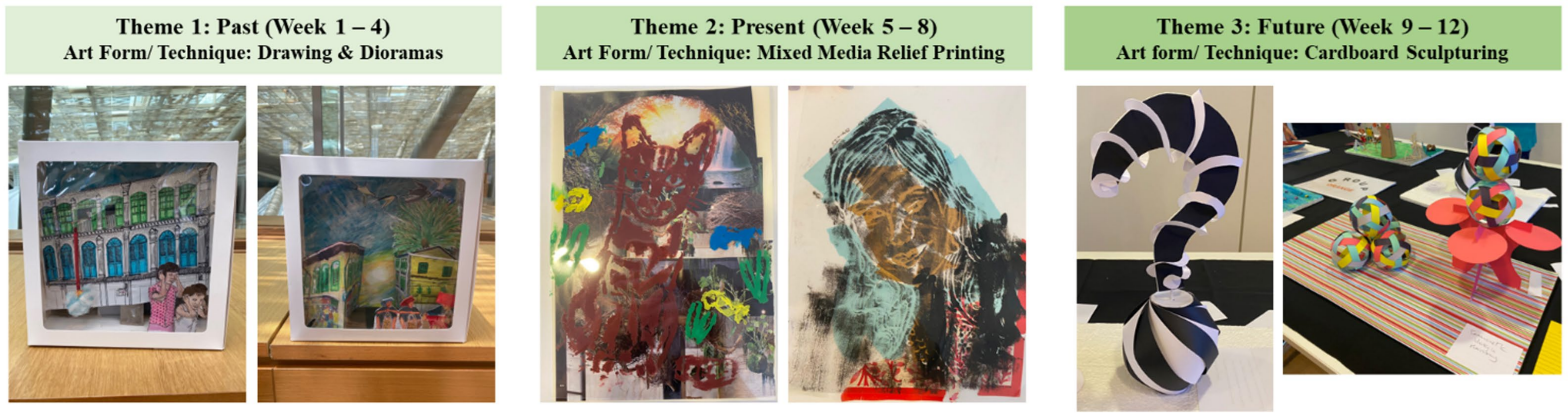
Samples of participants’ artworks.

Each domain comprised of four weekly sessions, 2 h per session. The first week involved a 45-min guided museum tour led by the museum docents, where the participants viewed and discussed selected artworks based on a thematic domain. This was followed by a 75-min, professional art educator-led artmaking session where participants were introduced to an artmaking technique and guided in developing their individual art pieces in response to the thematic domain. The subsequent three weekly sessions were dedicated to supporting the participants in realizing their creations with continued guidance from the art educator. The concluding session of each domain involved a showcase of the participants’ creations and a sharing of their ideas and meanings of their creation to the other participants. A summary of the intervention outline is detailed in Figure 3. At the end of the program, three art pieces, each responding to the thematic domains, were created by individual participants. Samples of participants’ artworks can be found in Figure 4.

The intervention was implemented at National Gallery Singapore, a renowned visual arts institution housing the world’s largest public collection of Singaporean and Southeast Asian modern art. The collaboration stems from a shared interest to explore and understand how cultural assets can foster wellbeing and human flourishing, coinciding in a timely manner with the museum’s endeavor to expand its role in the community by enhancing their community access program. The artworks used in the Singapore A-Health Intervention were paintings and sculptures by several key Singapore artists from the museum’s permanent collection (DBS Singapore Gallery). The decision to involve a range of representational and abstract art works was informed by the envisioned learning value of the program, aiming to expose participants to less familiar art forms and broaden their artistic horizons.

The artworks, topics for discussion, and art activities were developed jointly with the museum’s community access staff, art educators, and participants using a participatory action research method to encourage active engagement, relatability, inclusivity, ownership, and cultural specificity. The Singapore A-Health Intervention was subsequently implemented by museum staff, museum docents, professional art educator and members of the NTU research team. Specifically, the museum tours were conducted by the museum docents, while the artmaking component of the intervention was facilitated by the professional art educator and co-facilitated by research team members.

### 2.5 Pandemic-influenced protocol

As the study implementation coincided with the COVID-19 pandemic, nationwide health regulations affected the running of the study. Moreover, the target participants of this study, who were above age 60, were considered the “vulnerable population,” and additional safeguards were necessary to prevent infection. Regulations on permissible group sizes resulted in participants being divided into subgroups of three to eight and prevented them from mingling with participants from other subgroups. Moreover, lockdowns were implemented (27) over the course of the study resulting in segments of the program being run online. The subsequent introduction of vaccination differentiated measures (VDS) (28) prevented unvaccinated individuals from entering the museum, and a hybrid format with a mix of online and on-site implementation was adopted in those cases. For the workshops in phase one of the study, an online session was held on the twelfth week. As for the workshops in phase two of the study, the online sessions were held on the fourth to eighth week, while the hybrid sessions were held on the ninth to twelfth week.

An online intervention protocol based on the Singapore A-Health Intervention was developed as a contingency plan. The online intervention, which strictly adhered to the original protocol, allowed for a smooth transition between in-person sessions at the museum and online sessions in response to sudden changes in government directives. The online intervention was implemented on a secured video-conferencing platform, Zoom. User manuals and video tutorials were created and shared with participants, and assistance was provided to participants who required additional technical support. To enhance participant engagement on the online platform, breakout sessions were offered for personalized consultation by the art educator, and weekly milestones and lesson plans were sent to participants in dedicated chat groups prior to the sessions. The hybrid intervention was implemented on-site, with a dedicated research team member attending to the participants who participated in the program online. The art educator would first address the on-site participants and then the online participants. While the dedicated research team member was present to answer participant questions during the program, the art educator would also check in on the online participants to provide technical feedback. During the group sharing session, the research team member would project the video conference onto a projector so that the on-site participants could view the artworks of the online participants and would also share the artworks of the on-site participants with the online participants.

### 2.6 Outcome measures

Three outcome measures were adopted for this study. The first includes quality of life, as assessed by EuroQol-5D (EQ5D) which comprised of two parts (29). The first part is a questionnaire of five items on mobility, self-care, daily activities, pain, and depressive symptoms. Participants responded on a 5-point Likert scale where a lower score represented better quality of life. The second part of the questionnaire was a visual analog scale of the participant’s perceived health ranging from 0 (worst health) to 100 (best health one can imagine). The second outcome measure includes mental wellbeing, as assessed by Warwick-Edinburgh Mental Wellbeing Scales (WEMWBS), a 14-item scale assessing various domains of mental wellbeing (30). Participants responded on a 5-point Likert scale where a larger score represented better mental wellbeing. The third outcome measure include frailty scores as assessed by the Centre of Excellence on Longevity Self-administered Questionnaire (CESAM) (31). It is a 20-item scale assessing multiple aspects of health such as drug intake, memory complaints, health service utilization, and activities of daily living. The scores ranged from 0 (vigorous) to 18 (severe frail). Demographic information including age, gender, ethnicity, socio-economic status, and health status were collected from participants at baseline.

### 2.7 Data analysis

Allowing for an attrition rate of 5% at follow-up, a target sample of 110 (55 participants for each group) provides 90% power to detect an effect size of 0.55 (based on the results of the pilot study) between the intervention group and the control group at 5% level of significance (two-tailed test). Continuous variables were reported as mean and standard deviation (SD), and categorical variables were reported as frequencies and percentages. Linear mixed model was used (32) for repeated measures outcomes (EQ5D, CESAM, and WEMWBS) to derive estimates of average treatment effect across three follow-up time points with restricted maximum likelihood estimation and robust variance. Timepoint was specified as the fixed effect, and a random effect was specified for individuals to account for correlation between the repeated measurements on the same individual. The same model estimated differences between groups at the 5-, 9- and 12-week time points through modeling the interaction of treatment group and time (dummy). Mixed models are suitable for repeated measurements and allow the inclusion of all available data in the presence of dropouts (33). Regression coefficients (i.e., mean difference [MD]) and 95% confidence intervals (CIs) were estimated for the intervention group compared to the control group from the multivariable model and adjusted for age, sex, ethnicity, education, marital status, employment status, presence of chronic illness, number of workshops done, and baseline physical activity. Distribution of residuals was checked for the suitability of the fitted model and no concern was noted. All quantitative data were entered, and stored, in IBM SPSS v25 (Armonk, NY, USA). All analyses were conducted using Stata v18.0 (StataCorp, Taxes, USA) statistical software and two-sided *p* < 0.05 was considered for statistical significance. The Bonferroni correction (0.05/number of groups) was applied for pair-wise comparisons over time when interpreting statistical significance.

## 3 Results

### 3.1 Participant characteristics

A total of 112 participants were recruited, with 109 participants successfully completing the study. The age of participants ranged from 60 to 80 years (*M* = 66.6, *SD* = 3.80), were mostly female (77%), and of Chinese ethnicity (95%). The majority of the participants were retirees (63%), were physically active (95%), and half were living with a chronic health condition (54%). There was no significant difference for all demographic measures between the intervention and control group, except for age and physical activity. The intervention group participants attended an average of 11.25 (SD = 1.73) sessions of the Singapore A-Health Intervention. Please refer to Table 1 for the full participants’ information.

**Table 1:** Baseline characteristics of study participants.

Demographic information	Total (*n* = 112)	Control (*n* = 56)	Intervention (*n* = 56)
Age (year, mean (SD))	66.6 (3.80)	67.3 (4.05)	65.8 (3.40)
Sex (*n*, %)			
Male	26 (23.2)	11 (19.6)	15 (26.8)
Female	86 (76.8)	45 (80.4)	41 (73.2)
Marital status (*n*, %)			
Single	27 (24.1)	15 (26.8)	12 (21.4)
Married	73 (65.2)	36 (64.3)	37 (66.1)
Divorced / separated	7 (6.3)	3 (5.4)	4 (7.1)
Widowed	5 (4.5)	2 (3.6)	3 (5.4)
Highest Education Attained (*n*, %)			
GCE ‘N’, ‘O’ level, GCE ‘A’ Level or ITE/Higher Nitec and below	32 (28.6)	16 (28.6)	16 (28.6)
Polytechnic Diploma or Professional Certificate	20 (17.8)	11 (19.6)	9 (16.0)
Bachelor’s Degree	43 (38.4)	19 (33.9)	24 (42.9)
Postgraduate Degree	17 (15.2)	10 (17.9)	7 (12.5)
Ethnicity (*n*, %)			
Chinese	107 (95.5)	53 (94.6)	54 (96.4)
Indian	2 (1.8)	1 (1.8)	1 (1.8)
Other	3 (2.7)	2 (3.6)	1 (1.8)
Employment status (*n*, %)			
Full-time employed	9 (8.0)	3 (5.4)	6 (10.7)
Part-time employed	33 (29.5)	19 (33.9)	14 (25)
Unemployed / retired	70 (62.5)	34 (60.7)	36 (64.3)
Monthly income (*n*, %)			
No income	29 (25.9)	14 (25.0)	15 (26.8)
Less than S$1,500	29 (25.9)	17 (30.4)	12 (21.4)
S$1,500 to S$2,999	19 (17.0)	10 (17.9)	9 (16.1)
S$3,000 to S$4,999	9 (8.0)	6 (10.7)	3 (5.4)
S$5,000 to S$6,999	6 (5.4)	2 (3.6)	4 (7.1)
S$7,000 or more	5 (4.5)	1 (1.8)	4 (7.2)
Prefer not to tell	15 (13.4)	6 (10.7)	9 (16.1)
Presence of chronic illness (*n*, %)			
Yes	52 (46.4)	27 (48.2)	25 (44.6)
No	60 (53.6)	29 (51.8)	31 (55.4)
Polypharmacy (*n*, %)^a^			
None	55 (49.1)	23 (41.1)	32 (57.1)
One to four types of medication	53 (47.3)	32 (57.1)	21 (37.5)
Five to nine of medication	4 (3.6)	1 (1.8)	3 (5.4)
Home support (*n*, %)^b^			
Yes	1 (0.9)	1 (1.8)	–
No	111 (99.1)	55 (98.2)	56 (100)
ADL Score (/6, Mean ± SD)^c^	5.90 (0.328)	5.89 (0.366)	5.91 (0.288)
IADL Score (/4, Mean ± SD)^d^	3.97 (0.283)	3.95 (0.401)	4.00 (0)
Happy mood (*n*, %)^e^			
Happy	85 (75.9)	41 (73.2)	44 (78.6)
Unhappy	1 (0.9)	–	1 (1.8)
Neither one nor the other	26 (23.2)	15 (26.8)	11 (19.6)
Practice of physical activity (*n*, %)^f^			
Yes	107 (95.5)	51 (91.1)	56 (100)
No	5 (4.5)	5 (8.9)	–
History of falls in the past 12 months (*n*, %)^g^			
Yes	16 (14.3)	7 (12.5)	9 (16.1)
No	96 (85.7)	49 (87.5)	47 (83.9)
Number of A-Health Sessions Attended (Mean (SD))	–	–	11.25 (1.73)

^a^Taking prescribed medications for conditions; ^b^Receiving help from family, friend or professional for daily living activities; ^c^Ranging from 0 (dependent) to 6 (independent); ^d^Ranging from 0 (non-autonomous) to 4 (autonomous); ^e^Based on answer to the question “How do you feel today?”; ^f^Regular physical activity (walking, bicycle, etc.) at least one hour per week in the past month; ^g^Based on answer to the question “Did you fall in the previous year?”.

### 3.2 Outcome comparison results

Results from linear mixed models for each outcome are presented in Table 2. The mean overall quality of life improved over time in the intervention group compared to the control group. However, it was not statistically significant (MD −0.77, 95% CI −2.51 to 0.98, *p* = 0.390) with a similar pattern at each follow-up time. Similarly, there was no significant difference between intervention and control group (MD −10.24, 95% CI −28.17 to 7.68, *p* = 0.263) when quality of life analyzed on the visual analog scale.

**Table 2:** Summary of outcome measures by treatment group using linear mixed model.

Outcomes	Baseline	5-week post baseline		9-week post baseline		12-week post baseline	
	Mean (SD)	Mean (SD)	MD (95% CI)	Mean (SD)	MD (95% CI)	Mean (SD)	MD (95% CI)
Between group comparison							
EQ-5D							
Intervention	5.59 (1.04)	5.67 (1.06)	−0.03 (−0.31 to 0.24)	5.61 (0.96)	−0.12 (−0.47 to 0.23)	5.57 (0.90)	0.003 (−0.38 to 0.38)
Control	5.55 (1.04)	5.66 (0.86)		5.68 (0.81)		5.53 (1.10)	
EQ-5D VAS							
Intervention	85.9 (9.95)	84.7 (11.3)	−1.90 (−6.14 to 2.35)	86.3 (9.61)	0.47 (−3.93 to 4.87)	86.3 (9.42)	−0.54 (−4.96 to 3.89)
Control	85.3 (12.8)	85.9 (9.54)		84.9 (14.6)		85.9 (10.5)	
CESAM (Frailty)							
Intervention	1.46 (1.85)	1.65 (2.01)	−0.11 (−0.67 to 0.45)	1.02 (1.60)	−0.52 (−1.09 to 0.05)	1.15 (1.53)	−0.24 (−0.81 to 0.33)
Control	1.88 (1.50)	2.18 (2.09)		1.95 (1.80)		1.80 (1.73)	
Pre-test WEMWBS							
Intervention	57.7 (8.42)	57.7 (8.20)	0.05 (−1.60 to 1.71)	58.1 (8.02)	0.17 (−1.97 to 2.31)	58.6 (8.09)	−0.11 (−2.53 to 2.30)
Control	57.9 (6.78)	57.8 (6.35)		58.0 (6.43)		58.7 (7.63)	
Within intervention group only (pairwise) comparison							
CESAM (Frailty)	1.46 (1.85)	1.65 (2.01)	0.19 (−0.20 to 0.59)	1.02 (1.60)	−0.44 (−0.85 to −0.04)	1.15 (1.53)	−0.32 (−0.72 to 0.09)
Post-test WEMWBS	55.7 (7.18)	56.7 (8.50)	0.82 (−0.41 to 2.05)	57.6 (8.36)	1.65 (0.09 to 3.22)	58.5 (8.22)	2.42 (0.69 to 4.16)

VAS, Visual Analog Scale; WEMWBS, Warwick-Edinburgh Wellbeing Scale; SD, standard deviation; MD, mean difference; CI, confidence interval. Between group MD was calculated by the linear mixed model and each model was adjusted for age, sex, ethnicity, education, marital status, employment status, presence of chronic illness, number of workshops done, and baseline physical activity.

The overall mean frailty score showed some improvement over time in the intervention group compared to the control group, but it was not statistically significant (MD −1.17, 95% CI −4.20 to 1.86, *p* = 0.448) including at different follow-up times. Within the intervention group, a consistent significant improvement over time was observed (9-week vs. baseline MD −0.44, 95% CI −0.85 to −0.04, *p* = 0.032; 9-week vs. 5-week MD −0.64, 95% CI −1.03 to −0.24, *p* = 0.002; and 12-week vs. 5-week MD −0.51, 95% CI −0.91 to −0.10, *p* = 0.014).

Pre-test overall mean wellbeing score increased over time in the intervention group compared to the control group. However, it was not statistically significant (MD 1.47, 95% CI −12.05 to 15.00, *p* = 0.390) with similar pattern at each follow-up time. The post-test mean wellbeing score (in intervention group only) significantly improved over time at 9-week (MD 1.65, 95% CI 0.09 to 3.22, *p* = 0.039) and 12-week (MD 2.42, 95% CI 0.69 to 4.16, *p* = 0.006) compared to baseline scores.

## 4 Discussion

In summary, the quantitative findings indicated significant improvements over time in frailty and wellbeing for the intervention group but not quality of life. The findings of this study followed a similar trend as the original A-Health intervention in the Montreal Museum of Fine Arts (Quebec, Canada) where improvements in frailty, psychological wellbeing, and quality of life were found (21, 22, 34). In Asia, the A-Health intervention conducted in Tokyo Fuji Art Museum (Tokyo, Japan) resulted in significant improvements in quality of life but not wellbeing, as well as mixed results on frailty (35, 36). However, there were no interaction effects over time for all outcome measures in this study. Nonetheless, this study showcased the value of multi-sector collaboration, and showed the potential roles heritage institutions can play in supporting health promotion in Singapore (37). Furthermore, these findings add to the growing body of literature on the health promoting role of museum-based interventions (38–40). The current research also adds value by providing cross-cultural support and insights on the criteria for successful implementation of an art and museum-based program for healthy aging.

### 4.1 Understanding the results

The lack of interaction effects for the outcome measures could be due to the potential confounding factors due to the COVID-19 pandemic which impacted the findings. For instance, physical distancing measures required participants to be seated apart in sub-groups of three to eight. The enforcement of such rules may have limited the effectiveness of the program as participants had to be mindful of their interactions with others. The program coincided with two lockdowns, and this might have had an influence on the participant’s wellbeing and quality of life, which affected participants’ responses on the psychometric measures. There may also be latent effects of transitioning between an in-person intervention to an online or hybrid intervention which influenced the findings.

The significant effect of time on frailty and wellbeing suggests the potential effectiveness of the A-Health intervention in impacting the health of community dwelling older adults. Frailty is a reversible condition and determinants such as depressive symptoms, cognitive function, and the lack of social support (41) could be influenced by participatory arts engagement. Participation in a structured art and museum-based intervention offers an avenue for learning and skill development which could support cognitive functioning. Informal and formal lifelong learning at old age has been reported to be beneficial in the domains of mental, psychological, social, and physical health (42–44). For the Singapore A-Health Intervention specifically, the design of the program was intellectually stimulating and required the synthesis of new knowledge to form an art piece. For instance, the challenges presented in the program, (i.e., having to conceptualize an art piece within the confines of the given themes, while amalgamating contents from the museum tours and professional instruction) required one to activate multiple intelligences to solve hurdles and produce an art piece (45, 46). As a result, participants were driven to take ownership of their learning, and by the end of the program, they were observed to be more comfortable with ambiguity and adept in sourcing for information. To illustrate, participants initially reported that the lack of reference pieces for their artwork was a source of stress. However, for the subsequent art works, participants embraced the uncertainty and adapted by discussing with their peers or doing their own research. More information on the qualitative findings were reported elsewhere in this journal (47). Moreover, the program enabled participants to be more mindful and perceptive of their surroundings, which has benefits to one’s physical, mental, social and existential dimensions (48). The acquired skills are transferrable and could have a sustainable impact on the participant’s lives after the completion of the program (49). In addition, the group setting of the program supports social connections and relationship building within the community. With loneliness and social isolation on the rise among the older population (50, 51), the Singapore A-Health Intervention offers a safe and enriching way to remain connected even with the added restrictions during the pandemic. These positive impacts might have a virtuous effect on various domains of health, as explained by the multi-level theoretical framework of mechanisms of action which posits that there are multiple simultaneous causal mechanisms which interact with each other resulting in better outcomes (52). Overall, the current study contributes to the growing literature on the effectiveness of visual arts participation on mental and physical health benefits such as reduced chronic pain, increased mobility, and increased vigor among older adults (53).

### 4.2 Limitations and future directions

Despite the encouraging findings generated from this study, there are caveats that should be considered. Firstly, in terms of sampling, majority of the participants were female, of Chinese ethnicity and had a college degree which limits the generalizability of the findings. Additionally, participants were recruited through open recruitment where interested participants signed up for the study. It may be argued that the participants already had an interest in the arts prior to the intervention and this may be a confounder. Moreover, in the first phase of the study, spouses who were recruited were randomized into different groups (i.e., intervention and control group) and their informal exchange of experiences may have had an impact on the findings. However, this was addressed in the second phase where spouses were randomized into the same group. Future research could adopt a stratified sampling method for a more representative sample of the Singapore older population.

Secondly, this intervention was tested on a profile of healthy, community-dwelling older adults who were highly educated and English-speaking, and thus would require further refinement and testing for other populations of older adults. Also, while this study shown the positive impact of a 12-week, 24-h museum-based intervention, the optimal dosage is not ascertained. Future studies could investigate the suitable dosage of the A-Health intervention for social prescription.

Thirdly, the outcome measures used in this study consisted of self-reported measures which could be used as a foundation for further evaluation using a mixture of self-reported and objective health measures. Participants in the study also suggested that the quantitative measures might be limited in scope as it included only frailty, general wellbeing, and quality of life, and could be expanded to evaluate a more holistic assessment of health.

Fourthly, the intervals between assessment were relatively short (baseline, 5-weeks, 9-weeks, 12-weeks) and could be expanded in subsequent longitudinal investigations. Moreover, although efforts were made to conceal the allocation to avoid bias during the baseline assessment, researchers were aware of the group allocation after baseline assessment. As a result, there may be a possibility of an expectation bias, where improvements in the outcomes might be due to the participants’ expectations of benefit from receiving the treatment, rather than the treatment itself. While this is a challenge commonly faced in the field and reported in systematic reviews and meta-analyses (13, 17), future research could consider implementing the intervention with separate teams of interventionists and assessors to reduce bias. Furthermore, the control group was not wait-listed for later treatment, which may have led to nocebo effects. In future studies, participants in the control group could be assigned to an active group to reduce the risk of nocebo effects or a wait-list control group to ensure equal opportunities to receive the intervention. Researchers could also use measures to assess for the participant’s expectations about their assigned condition and control for these expectations during analysis.

Finally, as with other participatory arts interventions, the A-Health intervention is complex and multi-faceted. With external influences and further adjustments to the protocol due to the pandemic, replicability and generalizability was limited. Additionally, the intervention mechanisms were not ascertained in the current study. Nonetheless, implementation during the COVID-19 pandemic provided a unique opportunity to understand the impact of the program which shows promise in benefitting an older adult’s physical and psychological wellbeing even during a pandemic. Furthermore, this study highlighted the importance of a strong support system between stakeholders and structured session design for better adaptability during disruptions, and this aspect could be explored further in future studies. Future investigations could adopt other research approaches such as an implementation science approach or a realist approach for a holistic understanding of the intervention. Implementation science focuses on integrating research findings and evidence-based practices to enhance the quality and effectiveness of the intervention (54), while a realist approach could provide insights to how and for whom the intervention is effective for (55).

## 5 Conclusion

The Singapore A-Health intervention shows promise in enhancing wellbeing and improving frailty for community-dwelling older adults in Singapore during the pandemic. These findings support the 12-week A-Health protocol across cultures, adding to the growing body of empirical evidence on the benefits of arts and museum interventions, as well as the value of art and cultural institutions and multisector collaborations in supporting the health of rapidly aging populations. Similar findings from comparative international RCTs from Canada and Japan further show that such benefits transcend social, cultural, and societal contexts.

## Data availability statement

The datasets presented in this article are not readily available because the original contributions presented in the study are included in the article/supplementary material, further inquiries can be directed to the corresponding author. Requests to access the datasets should be directed to andyhyho@ntu.edu.sg.

## Ethics statement

The studies involving humans were approved by NTU Institutional Review Board (NTU-IRB). The studies were conducted in accordance with the local legislation and institutional requirements. The participants provided their written informed consent to participate in this study.

## Author contributions

AH and OB designed the study and obtained funding. MT designed the art and museum engagement activities. SM, SG, GY, AT, YY, and KG was involved in the implementation of the research study. AH, RB, and SM conducted the analysis. All authors contributed to data interpretation, as well as the writing and revision of the manuscript.
